# Safety evaluation of *FAD2* RNAi transgenic *Brassica napus* L. based on microbial diversity and metabonomic analysis

**DOI:** 10.3389/fpls.2022.953476

**Published:** 2022-12-01

**Authors:** Yanting Qi, Qiming Wang, Qingxuan Xie, Chuan Wu, Minhui Xu, Shaofan Han, Ting Zhou, Juan Li, Libing Xia, Wai chin Li, Weisong Pan

**Affiliations:** ^1^ College of Bioscience and Biotechnology, Hunan Agricultural University, Changsha, China; ^2^ School of Metallurgy and Environment, Central South University, Changsha, China; ^3^ Department of Science and Environmental Studies, The Education University of Hong Kong, Tai Po, Hong Kong SAR, China; ^4^ College of Agronomy, Hunan Agricultural University, Changsha, China

**Keywords:** safety evaluation, *FAD2* RNAi, *Brassica napus* L., microbial community, metabonomic

## Abstract

Oleic acid desaturase (*FAD2*) is the key enzyme that produces polyunsaturated fatty acids in rapeseed (*Brassica napus* L), which is one of the main oil crops. RNA interference (RNAi) is an emerging technique that provides new opportunities for the generation of new traits in plants. To increase oleic acid content and reduce linoleic and linolenic acid content in rapeseed, we constructed an ihpRNA plant expression vector of the *FAD2* gene and obtained transgenic plants for multiple generations by stable inheritance. In this study, third-generation transgenic plants (T3), seventh-generation transgenic plants (T7), and wild-type plants (WT) were used. The differences in microbial community diversity between transgenic plants and wild-type plants and the up- and downregulation of rhizosphere metabolite contents were investigated. In conclusion, the results showed that the soil microbial community structure was stable, the general microbial community structure was not changed by the transgenic rhizosphere exudates, and no significant harmful root exudate of transgenic rapeseed on the environment was found through the microbial community and metabolomics analysis. This work may provide an understanding of the impact of RNAi on plant metabolites and a safety evaluation method for transgenic plants and a reference for rapeseed breeding.

## Introduction

Rapeseed (*Brassica napus* L.) is one of the main oil crops in China and provides approximately 5.2 million tons of high-quality edible oil every year; rapeseed oil accounts for 47% of Chinese vegetable oil (Liu et al., 2019). Vegetable oil from rapeseed (*B. napus*) is one of the most commonly used vegetable oils in the world, and its oleic acid content is moderately high (approximately 65%) (Jung et al., 2011). Rapeseed oil contains high polyunsaturated fatty acid contents, such as linoleic acid and linolenic acid. These fatty acids are very unstable in terms of their chemical properties and are easily oxidized to form substances that are harmful to the human body. Oleic acid is a monounsaturated fatty acid with complex chemical properties. Studies have shown that oleic acid has good nutrition and healthcare functions by alleviating acute cadmium poisoning in mice (Chen et al., 2009; Yang et al., 2009; Guan et al., 2016; Wang et al., 2019). Therefore, reducing the content of polyunsaturated fatty acids, especially linolenic acid, and increasing the content of oleic acid have become important objectives of oil crop quality breeding (Sivaraman et al., 2004).

Oleic acid desaturase (*FAD2*) is the key enzyme for synthesizing polyunsaturated fatty acids in plants. *FAD2* is located in the endoplasmic reticulum (ER), and it is desaturated by catalysis of the formation of the double bond of oleic acid (18:1) at δ12 and plays a vital role in the formation of linoleic acid (18:2) (Lee et al., 2013). Seed oil with the largest change in fatty acid composition is produced by oil crops with high oleic acid, in which *FAD2* gene expression is inhibited (Lee et al., 2013).

RNA interference (RNAi) provides new opportunities to develop new traits in transgenic plants (Ramon et al., 2014). RNAi is an innovative gene-blocking technology, and RNAi has become a current functional genomic and genetic tool that is broadly applied to study gene functions by “knock-down” of cognate gene targets (Fishilevich et al., 2016). Compared with traditional transgenes, RNAi has the characteristics of high specificity, high efficiency, persistence, and signal conductivity, which can simply and efficiently inhibit the expression of specific genes. Therefore, RNAi has become an important field in the research and development of new transgenic plants. We found some studies on the ecological safety aspects of transgenic plants through a literature search in the early stage of the study (Chen et al., 2017; Cui et al., 2019; Tian et al., 2020). In regard to the application of genetically modified organisms (GMOs) in agriculture, discussions on this aspect have focused on consumer safety and the impact of genetically modified (GM) crops on the environment. Controlled on-site release and commercial applications of GMOs are regulated by risk assessment procedures in many countries. In the European Union, these procedures are guided by Directives 90/220/EEC and 94/15/EC (Pascher and Gollmann, 1999). China has not yet developed a special safety assessment guide for RNAi transgenic plants. Therefore, we can argue that if there is no change in the nutritional composition, nutritional value, and proposed use of GM crops, they can be considered basically equivalent to nongenetically modified crops (OECD, 1993). In addition, RNAi GM crops should be managed by classification according to the principle of case analysis and still need to be managed according to GM plants.

Comparing transgenic crops with their parents based on molecular characteristics is an effective method to evaluate the safety of transgenic crops, and it has been used in many studies on transgenic plants. Montero et al. (2011) found that approximately 0.40% of transcripts were differentially expressed in conventional rice varieties, and half of the difference between transgenic resistant rice and conventional rice was related to transgenic rice. Coll et al. (2009) compared widely used commercial GM-BT transgenic maize with nontransgenic varieties through transcriptome analysis, and they found differential expression of a small number of sequences in GM-BT maize. Wang et al. (2018) obtained stacked transgenic maize 12-5xIE034 that contained insecticidal *cry* and glyphosate tolerance *G10-epsps* genes by crossing of transgenic maize varieties 12-5 and IE034, and did transcriptome and metabolome analyses for different maize varieties; the results showed that the nine maize varieties had obvious differences in gene expression.

The microbial community is one of the important constituents of the ecosystem, and the long-term result of its influence is a change in soil quality. Root exudates of transgenic crops may have an impact on environmental safety because they interact with microorganisms in the soil, resulting in changes in microbial communities (Xiong et al., 2020). Therefore, it is necessary to study the change in soil microorganisms of transgenic *B. napus* L. under the dynamics of root exudates. Kremer et al. (2005) pointed out that the abundance of *Pseudomonas* in glyphosate-resistant soybean rhizosphere soil changed significantly. Means et al. (2007) studied the large-scale application of glyphosate and noted that transgenic glyphosate-resistant soybeans could have a significant impact on the composition and structure of soil microorganisms in this case. Saxena and Stotzky (2000) noted that planting transgenic crops did not cause significant changes in soil microorganisms. However, the soil environment is affected by various factors (Rajendran et al., 2019). In addition, the relationship between secretions from the roots of transgenic crops and microorganisms in the soil is also influenced by many study factors, such as the different materials used in the test; weather, moisture, and other environmental conditions during the test; and the artificial management. He et al. (2019) concluded that corresponding GM subrice and japonica rice had different effects on soil bacteria compared with their corresponding sister conventional rice. However, these differences in composition and abundance occurred only in a few genera and had no effect on the primary genera, and soil properties were mainly responsible for these differences. Khan et al. (2017) used 16S–23S rRNA intergenic spacer amplification to evaluate the genotypic diversity of soil microorganisms to assess the potential impact of transgenes, and they concluded that the NiC *B. napus* strain may not affect microbial enzyme activities and community structure in rhizosphere soils. The difference in varieties may be the reason for the slight variation in the test parameters. Lee et al. (2022) evaluated the effects of soybean (*Glycine max L*.) and hot pepper (*Capsicum annuum L*.) on soil microbial community structure, and the results showed that soil microbial communities were not significantly different between GM and non-GM strains. The results indicated that the soil microbial community structure was not significantly affected. Arshad et al. (2022) compared the rhizosphere bacterial populations, root exudates, and soil enzyme activities of nontransgenic and AVP1 genes in transgenic wheat. The results showed that the AVP1 gene in transgenic wheat had no obvious adverse effects on the soil environment and different bacterial communities. However, bacterial communities depend on several factors other than just the genetic makeup of the host plant.

In this study, to investigate whether RNAi transgenic plants have an impact on ecological environmental systems through changes in rhizosphere microbial diversity and metabolites, the soil samples and plant tissues of *FAD2* RNAi transgenic rapeseed were evaluated by using Illumina MiSeq sequencing technology and UPLC−MS/MS metabolomics technology. Comparative metabonomic analysis was conducted to obtain differential metabolites between transgenic rapeseed and the parent rapeseed, and the metabolic pathway changes caused by RNAi were also analyzed. This study provides a new basis for evaluating the safety of *FAD2* RNAi transgenic rapeseed from the molecular characteristics of metabolic terminals.

## Materials and methods

### Soil sample collection

*FAD2* RNAi transgenic *B. napus* L. was obtained through RNAi technology (Zhou et al., 2021b), and multiple generations of GM plants were obtained through stable inheritance. The phenotype of transgenic *B. napus L.* analyzed in this study and the specific planting and seed treatment procedures can be found in a previous study (Zhou et al., 2021b). T3, T7, and WT plants were used in this study (Zhou et al., 2021b). WT is wild-type *B. napus* L., and T3 and T7 are different generations of transgenic *B. napus* L., corresponding to the third generation and seventh generation, respectively. The two genotypes were provided by Jiangsu Academy of Agricultural Sciences (Zhou et al., 2021). Rapeseed plants were planted in a paddy field at Hunan Agricultural University, and the latitude and longitude were 28.18°CN and 113.08°CE, respectively. The sampling time was the summer season in 2018. We used a five-point sampling method. The soils for the experiments were collected at five points in each plot from the rapeseed field (1–30 cm depth) and mixed into one sample. The loose soil of the root system around the surface was shaken off the roots, and the soil that adhered strongly to the roots was carefully brushed from the roots and kept as rhizosphere soil. Stones and debris were removed manually and combined into one sample. Four composite soil samples were collected for each treatment, totalling 12 soil samples. Three treatments were designed, and each experimental treatment was replicated four times and repeated four times. Samples were stored at −80°C until pretreatment. Among the samples, Samples 1 to 4 were wild-type rapeseed (WT), Samples 5 to 8 were the third generation of *FAD2* RNAi transgenic *B. napus* L. (T3), and Samples 9 to 12 were the seventh generation of *FAD2* RNAi transgenic *B. napus* L. (T7).

### Microbial DNA extraction and illumina sequencing

Microbial community genomic DNA was extracted from the collected samples using the E.Z.N.A.^®^ soil DNA Kit (Omega Bio-Tek, Norcross, GA, USA) according to the manufacturer’s instructions. The hypervariable region ITS of the fungal gene was amplified with the primer pairs ITS3F (5’-GCATCGATGAAGAACGCAGC-3’) and ITS4R (5’-TCCTCCGCTTATTGATATGC-3’), and the hypervariable region V3–V4 of the bacterial 16S rRNA gene was amplified with the primer pairs 338F (5’-ACTCCTACGGGAGGCAGCAG-3’) and 806R (5’-GGACTACHVGGGTWTCTAAT-3’) (Toju et al., 2012; Liu et al., 2016). PCR reactions of the 16S rRNA gene and ITS gene, containing 25 μl of 2× Premix Taq (Takara Biotechnology, Dalian Co. Ltd., China), 1 μl of each primer (10 μM), and 3 μl of DNA (20 ng/μl) template in a volume of 50 µl, were amplified by thermocycling: 5 min at 94°C for initialization; 30 cycles of 30 s denaturation at 94°C, 30 s annealing at 52°C, and 30 s extension at 72°C; followed by 10 min final elongation at 72°C. The PCR instrument was Bio-Rad S1000 (Bio-Rad Laboratory, CA, USA). The PCR product was extracted from a 1% agarose gel, purified using the E.Z.N.A. Gel Extraction Kit (Omega, USA), according to the manufacturer’s instructions and quantified using a Quantus™ Fluorometer (Promega, USA) (Zuo et al., 2018). Sequencing libraries were generated using NEBNext^®^ Ultra™ II DNA Library Prep Kit for Illumina^®^ (New England Biolabs, MA, USA) and index codes were added. The library quality was assessed on the Qubit@ 2.0 Fluorometer (Thermo Fisher Scientific, MA, USA). Lastly, purified amplicons were pooled in equimolar amounts and paired-end sequenced on an Illumina Nova6000 platform and 250-bp paired-end reads were generated (Guangdong Magigene Biotechnology Co., Ltd. Guangzhou, China).

### Metabolite extraction and metabolomic analysis

Fifty milligrams of soil sample was accurately weighed, and the metabolites were extracted using a 400-µl methanol:water (4:1, v/v) solution. The mixture was allowed to settle at −20°C and treated by a high-throughput tissue crusher (Wonbio-96c, Shanghai Wanbo Biotechnology Co., Ltd) at 50 Hz for 6 min, followed by vortexing for 30 s and ultrasound at 40 kHz for 30 min at 5°C. The samples were placed at −20°C for 30 min to precipitate proteins after centrifugation at 4°C for 15 min. The supernatant was transferred to sample vials for LC−MS/MS analysis (Wang and Xie, 2020).

Multivariate statistical analysis was performed by using the ropls R package (Version 1.6.2, http://bioconductor.org/packages/release/bioc/html/ropls.html). Principal component analysis (PCA) using an unsupervised method was applied to obtain an overview of the metabolic data. All metabolite variables were scaled to unit variances prior to conducting the PCA. Partial least squares discriminate analysis (PLS-DA) was used for statistical analysis. All metabolite variables were scaled to Pareto scaling prior to conducting the PLS-DA. The model validity was evaluated from model parameters *R* ^2^ and *Q* ^2^, which provide information for interpretability and predictability, respectively, and avoid the risk of overfitting (Wang et al., 2021). Variable importance in the projection (VIP) was calculated in the PLS-DA model, and *p*-values were estimated with paired Student’s *t*-test on single-dimensional statistical analysis. The variables with significant differences of *p* < 0.05 and VIP values > 1 were defined as key metabolites.

The raw 16S rRNA gene-sequencing reads and raw ITS gene-sequencing reads were demultiplexed, quality-filtered by fastp Version 0.20.0, and merged by FLASH Version 1.2.7. Operational taxonomic units (OTUs) with a 97% similarity cutoff were clustered using UPARSE Version 7.1 (Edgar, 2013), and chimeric sequences were identified and removed. QIIME software and R language tools were used to analyze the community structure of samples at different classification levels. The taxonomy of each OTU representative sequence was analyzed by RDP Classifier Version 2.2 against the 16S rRNA database (silva132/16s_bacteria) and ITS database (unite7.2/its_fungi) using a confidence threshold of 0.7 (Wang et al., 2007).

### Statistical analysis

Statistical analyses were performed by SPSS 19.0 and summarized as the means ± standard errors (SE). The level of significance of various treatments was set at *p* < 0.05 using one-way analysis of variance (ANOVA).

## Results and discussion

### Microbial composition of the rhizosphere microbial community of wild-type and transgenic rapeseed plants

**Figure 1:**
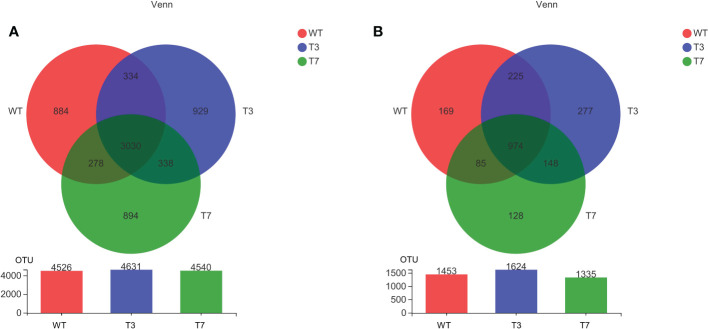
Venn diagram of the microbial community at the OTU level of wild-type and transgenic rapeseed plants (**A**, bacteria; and **B**, fungi). Note: The number of overlapping parts represents the number of species common to multiple groups, and the number of nonoverlapping parts represents the number of species unique to the corresponding group.

**Figure 2:**
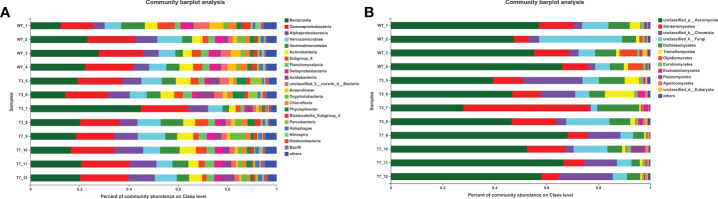
Percent of community abundance at the class level of wild-type and transgenic rapeseed plants (**A**, bacteria; and **B**, fungi). Note: The different colors of the column represent different species, and the length of the column represents the size of the proportion of the species.

The number of common and specific OTUs of groups is displayed in **Figure 1** to show the overlaps of OTUs among the rhizosphere soil from WT, T3, and T7 plants. The OTUs of bacteria and fungi totalled 4,526 and 1,453 in the rhizosphere soil of WT, respectively. The OTUs of bacteria and fungi totalled 4,631 and 1,624 in the rhizosphere soil of T3, respectively, and the OTUs of bacteria and fungi totalled 4,540 and 1,335 in the rhizosphere soil of T7, respectively. Compared with that of the WT, the number of genera in the root soil of T7 decreased relatively, while the number of genera in T3 increased relatively. The number of common OTUs of bacteria and fungi in root soil was 4,004 among WT, T3, and T7, representing 66.97%, 64.01%, and 68.15% of the total number of OTUs in WT, T3, and T7, respectively. The composition of the microbial communities of WT, T3, and T7 is shown in **Figure 2**. There was rarely any difference in rhizosphere microbial abundance between wild-type and transgenic plants. *Bacteroidia*, *Gammaproteobacteria*, *Ascomycota*, and *Sordariomymycetes* were found in high proportions in each sample. Moreover, *Ascomycota* accounted for a very high proportion of the fungal communities. In addition, the abundance of *Bacteroidia* in sample No. 7 of T3 (T3_7) was as high as 44%, and the abundance of *Sordariomymycetes* in this sample was also high. There was no difference in the composition of microbial species at the class level in the samples, but the abundance of microbial species was slightly different. Zhou et al. (2021a) found that Bt corn residues had no direct effect on soil bacterial communities compared with the decomposition time and environment. The functional diversity and metabolic activity of the microbial community indicated that there was no significant difference between Bt maize IE09S034 and the control group. A study found differences in microbial community structures between Bt and non-Bt maize fields, and there were also variations between the chemical and biochemical properties of rhizosphere soils under Bt and non-Bt maize cultivation; they concluded that these differences could be related to agricultural practices and varieties (van Wyk et al., 2017).

**Figure 3:**
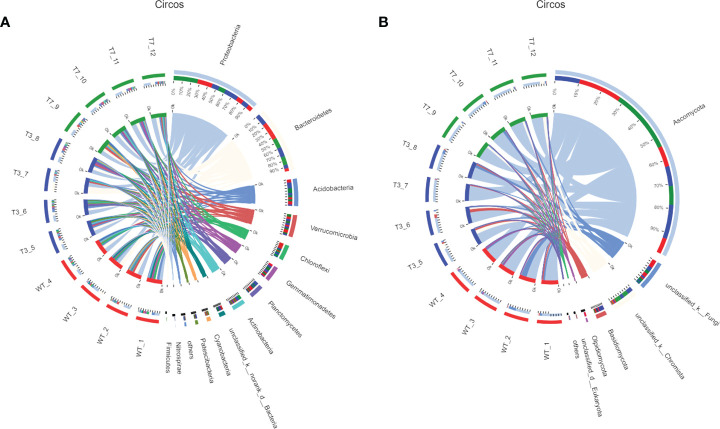
Circos diagram of bacteria and fungi at the genus level in different generations (**A**, bacteria; **B**, fungi). Note: The small semicircle (left half circle) represents the species composition in the sample. The large semicircle (right half circle) represents the distribution ratio of species in different samples at the taxonomic level.

In **Figure 3**, the dominant species with high abundance at the genus level of rhizosphere bacteria in wild-type and transgenic plant were *Proteobacteria* and *Bacteroidia*. At the genus level of rhizosphere fungi in wild-type and transgenic plant, the dominant species was *Ascomycota*. They had a high percentage in all samples. All other soil samples of T3, T7, and WT at the genus level showed very small differences in microbial abundance. As shown in **Figure 3**, the abundance of *Proteobacteria, Bacteroidetes, Acidobacteria, Verrucomicrobia, Chloroflexi*, and *Ascomycota* was relatively high at the genus level. Some studies have used *Proteobacteria* and *Acidobacteria* as indicators of land use change and soil pollution (Schneider et al., 2017; Kim et al., 2021). Members of the class *Bacteroidia* are Gram-negative, non-spore-forming, mostly anaerobic bacteria that decompose organic substances, and they thrive in diverse environments, such as soils and subsurface sediments (Garcia-Lopez et al., 2019; Podosokorskaya et al., 2020). *Ascomycota* is by far the largest group in the fungal kingdom. Ecologically important mutualistic associations, such as mycorrhizae and lichens, have evolved in this species and are regarded as pivotal innovations that supported the evolution of land plants (Beimforde et al., 2014). These results showed that the soil microbial community structure was stable, and the general microbial community structure of rhizosphere soil was not changed by the transgenic plants. Zou et al. found that 5-year-old transgenic Poplar 741 could not cause ecological risk and could not affect the microbial community structure or functional diversity (Zou et al., 2018). Fan et al. (2020) analyzed the effects of transgenic poplar on related bacterial communities from various aspects, including bacterial OTU composition, alpha and beta diversity, gene function, and differences among groups. Overall, transgenic poplar did not significantly affect endophytic or soil bacteria. Our results on the structural composition and diversity of the microbial community are consistent with these studies. Therefore, we preliminarily concluded that transgenic *B. napus* L. had no harmful effects on microbial communities in the environment.

### Alpha diversity analysis of the rhizosphere microbial community of wild-type and transgenic rapeseed plants

**Figure 4:**
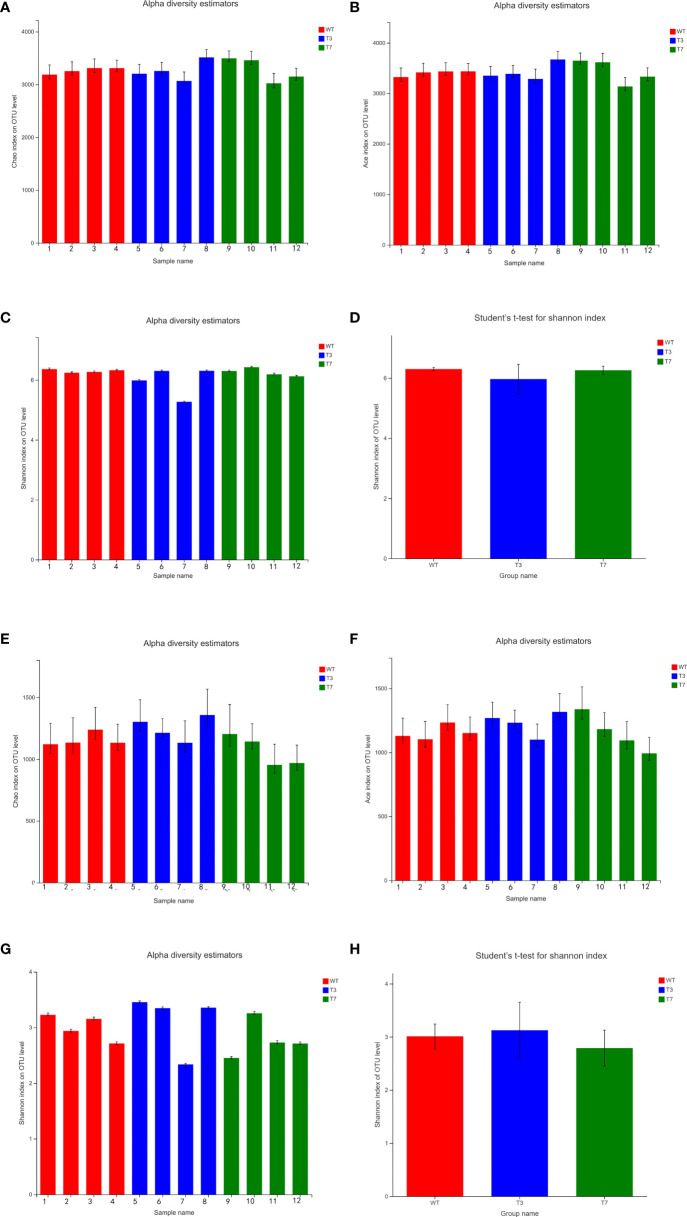
Alpha diversity index bar graph and alpha diversity significance difference test of interrooted prokaryotic and eukaryotic microbes (**A–D**, bacteria; **E–H**, fungi). Note: Student’s *t*-test was used to calculate the difference between the two groups, and the two groups were marked with significant differences (0.01 < *p* ≤ 0.05 was marked with *, 0.001 < *p* ≤ 0.01 was marked with **, and *p* ≤ 0.001 was marked with ***).

Alpha diversity refers to the diversity in a specific region or ecosystem. The commonly used indicators to characterize alpha diversity are Chao, ACE, and Shannon. The ACE index, Chao index, and Shannon index were obtained at a 97% similarity level using Mothur software. The Shannon index measures the average degree of uncertainty in predicting to what species a randomly selected individual from a set of S species and N individuals will belong to, and the value increases as the number of species increases and individuals become evenly distributed among species (Pylro et al., 2014). The ACE index and Chao1 index reflect the number of species in the community. **Figure 4** shows the alpha diversity analysis of the microbial community and significance difference test. The results of some samples showed fluctuations but few changes within a certain range, and the diversity indicators of WT, T3, and T7 were close to each other. Compared with transgenic plants and wild-type plants, the ACE index value and Chao index showed little difference between transgenic plants and wild-type plants, indicating that the transgenic plants did not cause obvious changes in the richness of the bacterial community. The Chao index of the fungal community from T7 was slightly smaller than that of WT and T3, indicating that the fungal community had higher richness in WT and T3. By comparing the two indicators, no obvious difference in bacterial community diversity between transgenic plants and wild-type plants was found, while the fungal community diversity of T7 was slightly smaller than that of the WT and T3. Overall, there was no significant difference in the alpha diversity of the fungal community and bacterial community.

Zhang et al. (2015) detected no OsrHSA protein in the roots of OsrHSA transgenic rice and concluded that OsrHSA transgenic rice and the rHSA protein produced by transgenic rice did not change the functional diversity of rhizosphere microbial communities. Another study also confirmed that microbial community diversity was not significantly influenced by the presence of GM plants (Riglietti et al., 2006). Our results were similar to these studies. In summary, through RNAi technology, *B. napus* L. had no obvious effect on the richness and diversity of the rhizosphere bacterial community and fungal community.

### Microbial abundance analysis of the rhizosphere microbial community of wild-type and transgenic rapeseed plants

**Figure 5:**
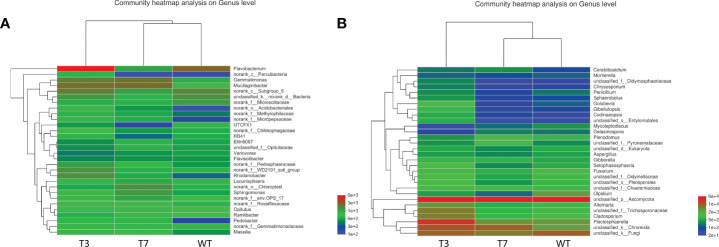
Heatmap of microbial communities at the genus level of wild-type and transgenic rapeseed plants (**A**, bacteria; **B**, fungi). Note: The variation in the abundance of different species in the sample is displayed through the color gradient of the color block.

As shown in **Figure 5**, the heatmap graph color represents the size and proportion of data in a two-dimensional matrix or table, thus showing the composition and structure of the microbiome and reflecting the similarity and differences of the community composition of different groups or different samples at each classification level. In the heatmap of bacterial and fungal abundance, the abundance of each microbial species in WT, T3, and T7 was similar. *Flavobacterium* in T3 was significantly more than that in WT and T7, and *Ascomycota* was abundant in WT, T3, and T7, which was consistent with the above analysis. The dominant genus and proportion between transgenic rapeseed plants and wild plants were consistent with the structural analysis at the genus level. A study found that compared with the parental line, the transgenic strain had no obvious effect, and for all evaluated microbial communities, soil type and the year of planting maize were the dominant factors affecting their structures, and no difference in the total number of bacteria between the rhizospheres of GM and parental plant lines was observed (Saxena and Stotzky, 2001). Chun et al. (2012) found no difference between transgenic rice and its parental nontransgenic counterpart (cultivar Dongjin) in the diversity index and community composition of the microbial community. In contrast, this change in the microbial community depended strongly on the growth stage and year. Therefore, they did not observe the adverse effects of these transgenic rice crops on the microbial community in paddy soils.

### Comparative analysis of metabolites in wild-type and transgenic rapeseed plants

**Figure 6:**
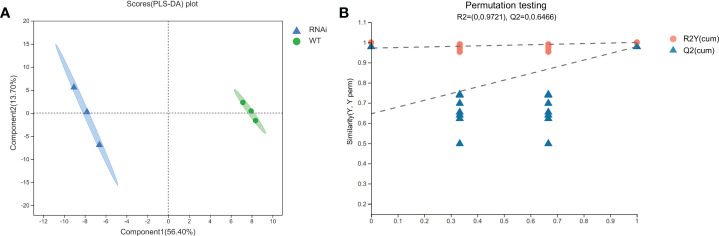
PLS-DA scoring models **(A)** and permutation test **(B)** of wild-type and transgenic rapeseed plants. Note: Component 1 denotes the first principal component interpretation degree, and Component 2 denotes the second principal component interpretation degree. The contribution rate of Principal Component 1 is 56.40%, and the contribution rate of Principal Component 2 is 13.70%.

PLS-DA can maximize the intergroup distinction and is conducive to finding differential metabolites, and it belongs to beta diversity approaches. PLS-DA obtained two principal components: the contribution rate of Principal Component 1 was 56.40%, the contribution rate of Principal Component 2 was 13.70%, *R* ^2^X = 0, *R* ^2^Y = 0.9721, and *Q* ^2^ = 0.6466. PLS-DA was verified for alignment (*n* = 200, i.e., 200 alignment experiments). In the model verification (**Figure 6B**), the horizontal line corresponds to *R* ^2^Y and *Q* ^2^ of the original model, and the red dot and blue dot represent *R* ^2^Y’ and *Q* ^2^’ of the model after replacement of y, respectively. *R* ^2^Y’ and *Q* ^2^’ were both smaller than *R* ^2^Y and *Q* ^2^ of the original model; i.e., the corresponding points did not exceed the corresponding lines, which indicated that the model was meaningful and that its differential metabolites could be screened according to VIP value analysis. According to the PLS-DA model (**Figure 6A**), 726 metabolome data points were analyzed. The samples of RNAi-T3 were distributed on the left side of the confidence interval, and the samples of WT were distributed on the right side of the confidence interval. The distinguishing effects of the two samples were abundant and obvious.

The root exudates of rapeseed mainly included alcohols, hydrocarbons, acids, and esters, and the contents of alcohols and hydrocarbons were relatively high. Hydrocarbons, esters, and acids or secondary metabolites have certain chemical influences, including the inhibition or stimulation of seed germination in other crops and an influence on microorganisms in the soil, in keeping with the results of Yang (2006).

### Differential metabolite analysis of wild-type and transgenic rapeseed plants

**Figure 7:**
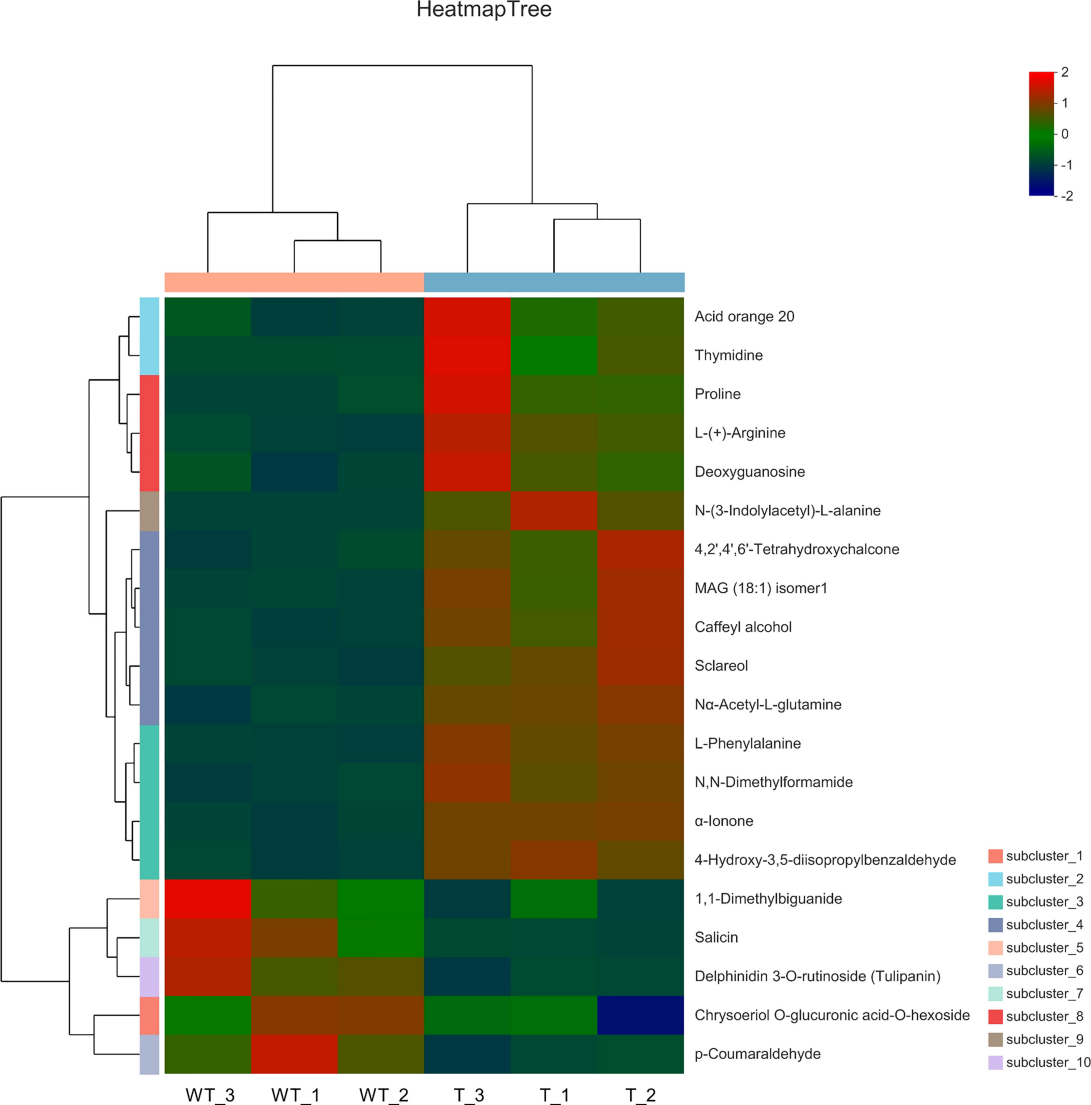
Heatmap of metabolic differences of the top 20 substances between wild-type and transgenic rapeseed plants. Note: Metabolites with fold change ≥2 or fold change ≤0.5 have significant differences.

Comparing the multiple changes in quantitative information for metabolic components in WT and RNAi samples, log2 treatment was carried out on the difference multiple. The top 20 differentially expressed metabolic components with changes are shown in **Figure 7**. Compared with WT plants, five types of amino acids and their derivatives [proline, L-(+)-arginine, N-(3-indolylacetyl)-L-alanine, Nα-acetyl-L-glutamine, and L-phenylalanine], one aldehyde and its derivatives (4-hydroxy-3,5-diisopropylbenzaldehyde), one amide and its derivatives (N,N-dimethylformamide), one chalcone (4,2’,4’,6’-tetrahydroxychalcone), one alcohol and its derivatives (caffeyl alcohol and sclareol), one ketone and its derivatives (α-lonone), one glycoside and its derivatives (deoxyguanosine), one pyrimidine and its derivatives (thymidine), and two others [acid orange 20 and MAG(18:1) isomer1] increased in relative content in RNAi-T plants. However, one guanidine and its derivatives (1-dimethylbiguanide), one polyether and its derivatives (salicin), one alcohol and its derivatives (chrysoeriol), two glycosides and their derivatives [O-glucuronic acid-O-hexoside and delphinidin 3-O-rutinoside (Tulipanin)], and one aldehyde and its derivatives (1 p-coumaricaldehyde) were reduced in relative content in RNAi-T plants.

Based on the PLS-DA results, the VIP of the PLS-DA model was analyzed from the obtained multivariate analysis. A total of 88 upregulated differential metabolites were detected, namely, 19 amino acids and their derivatives; 15 organic acids and their derivatives; 14 lipids; 6 nucleotides and their derivatives; 5 flavanones; 4 alkaloids; 3 phenylpropanoids, flavonoids, and flavonols; 2 alcohols, polyphenols, and procyanidins; 1 phenolamine, terpenoid, and vitamin and their derivatives; 1 indole and its derivatives; and 6 other metabolites. A total of 24 downregulated differential metabolites were detected, namely, 10 phenylpropanoids, 3 lipids, 3 flavonols, 2 polyphenols, 2 organic acids and their derivatives, 1 amino acid and its derivatives, 1 phenolamine, 1 nucleotide and its derivatives, and 1 vitamin. A volcano map was used to visualize the differential data for observation and analysis. It was found that there were both upregulated and downregulated substances with significant differences. Metabolites with upregulated expression were more abundant than metabolites with downregulated expression. However, there was no significant change in the expression of most metabolites.

**Figure 8:**
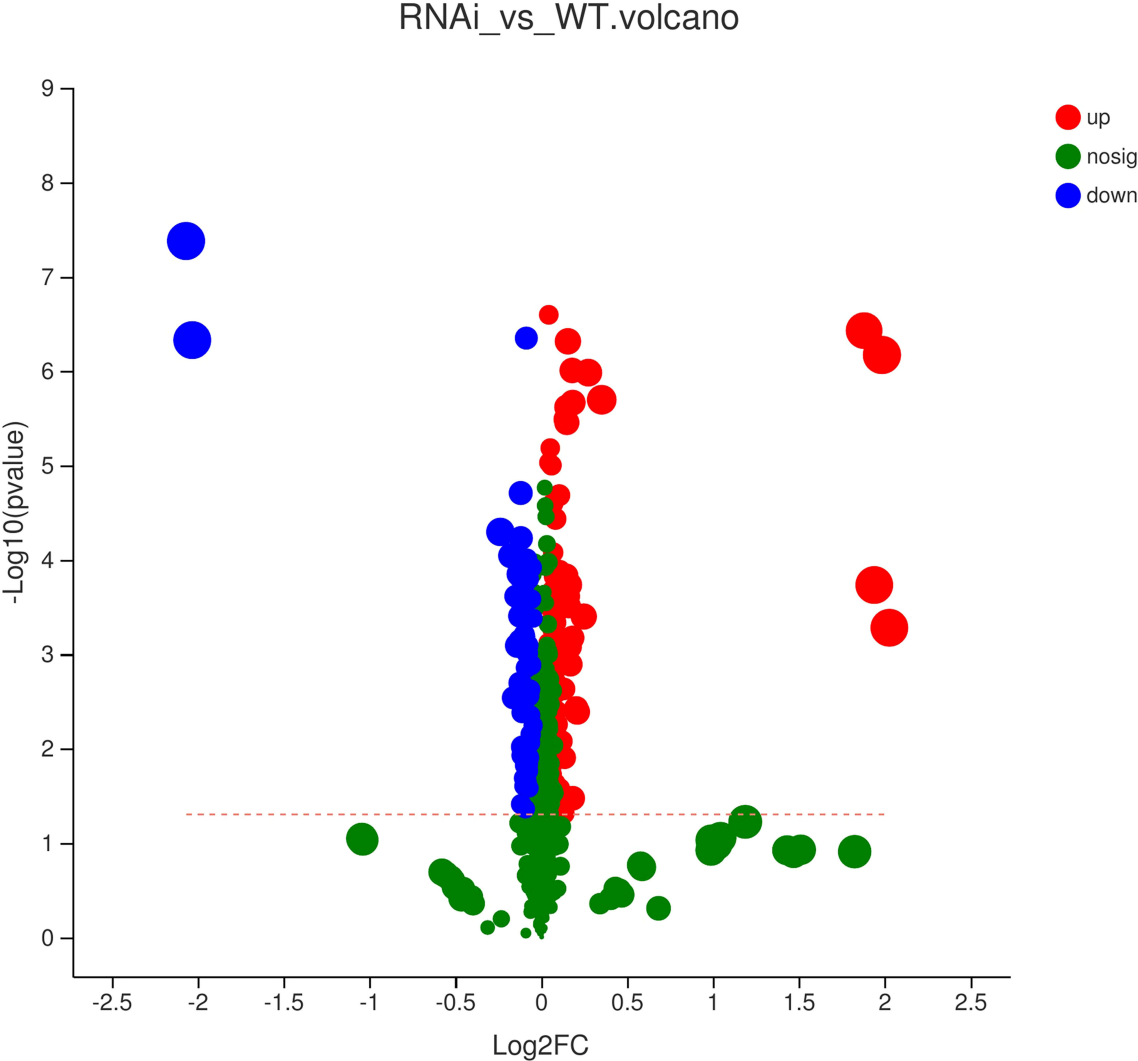
Volcano map of metabolic differences of wild-type and transgenic rapeseed plants (RNAi vs. WT). Note: Each dot in the figure represents a specific metabolite, and the size of the dot indicates the VIP value (the importance of the independent variable *x* in explaining the dependent variable *y*). The blue dots on the left are differentially downregulated metabolites, and the red dots on the right are differentially upregulated metabolites. The more dots on the left and right there are, the more significant the differences, and the higher the ordinate value is, the more significant the expression difference.

All metabolites were analyzed by *t*-test and two-tailed test, and the differences in metabolites were plotted (**Figure 8**). A point represented a metabolite, and a horizontal coordinate represented a pair of values. As shown in **Figure 8**, only a few metabolites showed significant differences. The expression levels of more differential metabolites increased in GM rapeseed than in wild-type rapeseed, and the expression levels of some metabolites were reduced. Specifically, there were six metabolites with significantly upregulated and downregulated changes. The main metabolites significantly upregulated were quinic acid O-glucuronic acid, N-(3-indolylacetyl)-L-alanine, L-tyramine, and phenylacetyl-L-glutamine. There were two significantly downregulated differential metabolites, homovanilloyl quinic acid and N-caffeoyl agmatine. It was necessary to demonstrate that the presence of these metabolites and expressed changes were not harmful to the environment based on relevant studies. Alanine is an amino acid with a neutral charge that can be sorbed into soil particles by (1) cation exchange, (2) anion exchange, or (3) ligand exchange (Apostel et al., 2017). Alanine is also the richest amino acid in soluble organic substances (Fischer et al., 2007). Therefore, this kind of metabolite is common in the environment. In addition, a study found that feruloyl tyramine, feruloyl tryptamine, and feruloyl dopamine were effective against *S. aureus* 209 and *S. pyogenes*, and the MIC was between 190 and 372 μM (Georgiev et al., 2012). Glutamine affected the level of other amino acids utilized by microorganisms, and the addition of glutamine reduced (P<0.05) the use of alanine and glycine by *E. coli* while stimulating (P<0.05) the use of threonine in the bacteria (Dai et al., 2013). Aromatic compounds are produced by microorganisms in the synthesis of the aromatic components of their own cellular materials. In addition to these anabolic syntheses, some microorganisms produce aromatic metabolites in the process of oxidative assimilation of nonaromatic materials. Quinic acid (1,3,4,5-tetrahydroxycyclohexanecarboxylic acid) was attacked by Micrococcus chinicus, resulting in protocatechuic acid (Rogoff, 1958). In *Neurospora crassa* and *Aspergillus nidulans*, the exogenous supply of quinic acid as a growth carbon source led to the production of three enzymes that were essential for the catabolism of quinic acid into protocatechuic acid (Watts et al., 2002). Such acids can be used by microorganisms and do not harm the environment. Polyamines play a variety of roles in every living organism, including physiological responses to pathogens in plants (Jiménez-Bremont et al., 2014). In fungi, they are involved in metabolism, regulation (Valdés-Santiago et al., 2012), and stress-coping functions (Valdés-Santiago and Ruiz-Herrera, 2014), and they also do not cause harm to the environment. In conclusion, the metabolites with variable expression levels were not harmful to the environment.

## Conclusion

In summary, the microbial community analysis showed that transgenic plants caused no significant changes in microbial (both bacterial and fungal) community composition and did not cause obvious changes in microbial community diversity, which indicated that transgenic *B. napus* L. might have no specific impacts on the rhizosphere microbial community abundance in rhizosphere soils and no risk to the environment. Root exudates were either upregulated or downregulated in transgenic *B. napus* L., and there were six differential metabolites with very significant changes. The main differential metabolites significantly upregulated were quinic acid O-glucuronic acid, N-(3-indolylacetyl)-L-alanine, L-tyramine, and phenylacetyl-L-glutamine. The main significantly downregulated differential metabolites were homovanilloyl quinic acid and N-caffeoyl agmatine. Relevant studies have shown that none of these metabolites are harmful to the environment. Therefore, based on the research results, we can basically infer that no harm to the ecological environment based on the dynamics of microbial diversity and metabonomic analysis was detected in transgenic plants cultivated in fields. Therefore, transgenic rapeseed has the potential for application. However, its safety needs to be further evaluated. Therefore, the future assessment of ecological safety in transgenic plants needs further study.

## Data availability statement

The data presented in the study are deposited in the NCBI repository, accession number SRR17344596-SRR17344612.

## Author contributions

The overarching research goals were developed by WP, QW, and YQ carried out experiments and conducted the first draft of the manuscript. WL guided the study and reviewed the manuscript. CW reviewed the manuscript and proposed some important advice. YQ: Experiment; Data curation; Formal analysis; Software; Visualization; Writing - review & editing. QW: Experiment; Data curation; Writing - review & editing. CW: Methodology; Resources; Writing - review & editing. WP: Funding; Conceptualization; Resources. QX: Experiment; Data curation; Writing - review & editing. MX: Experiment; Data curation. SH: Experiment; Data curation. TZ: Experiment; Data curation. LX: Experiment; Data curation. JL: Review & editing. WL: Review & editing. All authors contributed to the article and approved the submitted version.

## Funding

Financial support from the Research Foundation of Education Bureau of Hunan Province, China (No. 20A246), the Natural Science Foundation of Hunan Province of China (No. 2021JJ30340), the National Natural Science Foundation of China (No. 42177392), and the Dean’s Research Fund [Project code: 04626 (2020/21) and 04730 (2021/22)] of the Education University of Hong Kong is gratefully acknowledged.

## Conflict of interest

The authors declare that the research was conducted in the absence of any commercial or financial relationships that could be construed as a potential conflict of interest.

## Publisher’s note

All claims expressed in this article are solely those of the authors and do not necessarily represent those of their affiliated organizations, or those of the publisher, the editors and the reviewers. Any product that may be evaluated in this article, or claim that may be made by its manufacturer, is not guaranteed or endorsed by the publisher.
